# Supplementary material for: Ameliorative effects of elderberry (Sambucus nigra L.) extract and extract-derived monosaccharide-amino acid on H2O2-induced decrease in testosterone-deficiency syndrome in a TM3 Leydig cell
Source: PLoS One. 2024 Apr 25;19(4):e0302403. doi: 10.1371/journal.pone.0302403 (PMC11045058; doi:10.1371/journal.pone.0302403)
Supplement: S1 Fig — (DOCX) [file pone.0302403.s001.docx]

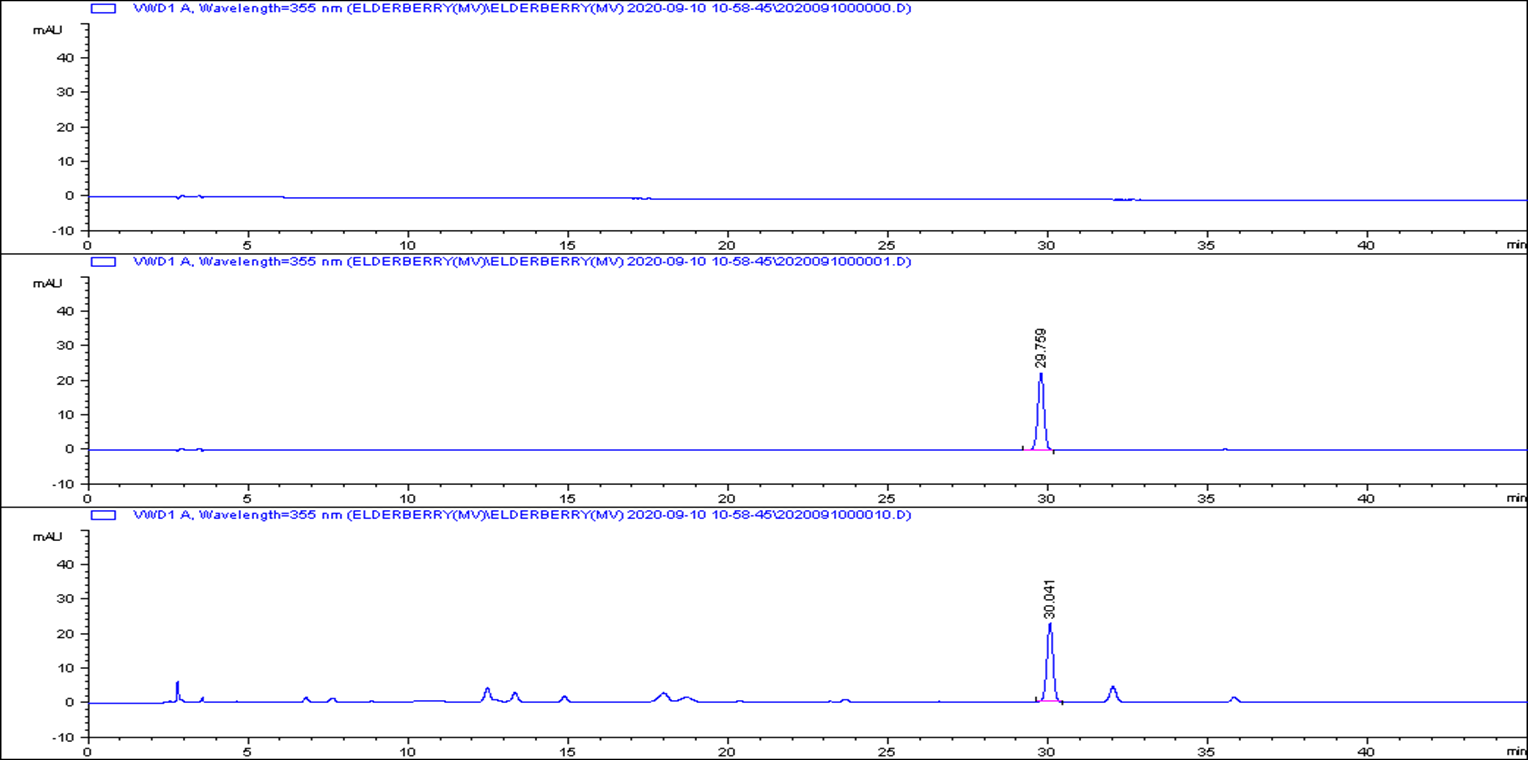


Blank

STD

Elderberry ext.

**Rutin**

**S1 Fig. HPLC analysis of the establishment of rutin as an indicator in the extracts of elderberries.** This test was conducted by the korea functional food research center. STD: Rutin standard(PHL-89270, Sigma Aldrich, CAS 153-18-4)
