# Supplementary material for: Ameliorative effects of elderberry (Sambucus nigra L.) extract and extract-derived monosaccharide-amino acid on H2O2-induced decrease in testosterone-deficiency syndrome in a TM3 Leydig cell
Source: PLoS One. 2024 Apr 25;19(4):e0302403. doi: 10.1371/journal.pone.0302403 (PMC11045058; doi:10.1371/journal.pone.0302403)
Supplement: S2 Fig — A: Analysis of elderberry extract by liquid chromatography/mass spectrometry. B: Analysis of elderberry extract by multiple reaction monitoring. FL, fructose–leucine. (DOCX) [file pone.0302403.s002.docx]

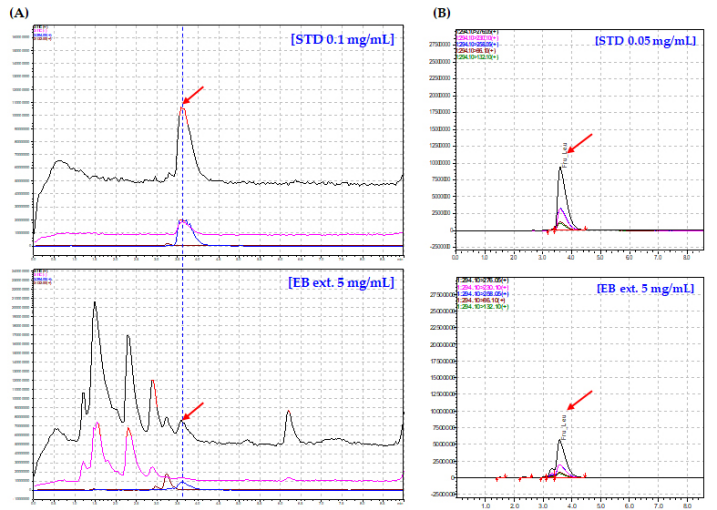


**S2 Fig. Analysis of FL of elderberry extract.** A: Analysis of elderberry extract by liquid chromatography/mass spectrometry. B: Analysis of elderberry extract by multiple reaction monitoring. FL, fructose–leucine (SC-470657A, Santa Cruze Biotechnology Inc., CAS 34393-18-5)
