# Supplementary material for: Ameliorative effects of elderberry (Sambucus nigra L.) extract and extract-derived monosaccharide-amino acid on H2O2-induced decrease in testosterone-deficiency syndrome in a TM3 Leydig cell
Source: PLoS One. 2024 Apr 25;19(4):e0302403. doi: 10.1371/journal.pone.0302403 (PMC11045058; doi:10.1371/journal.pone.0302403)
Supplement: S3 Fig — FL, fructose–leucine. (DOCX) [file pone.0302403.s003.docx]

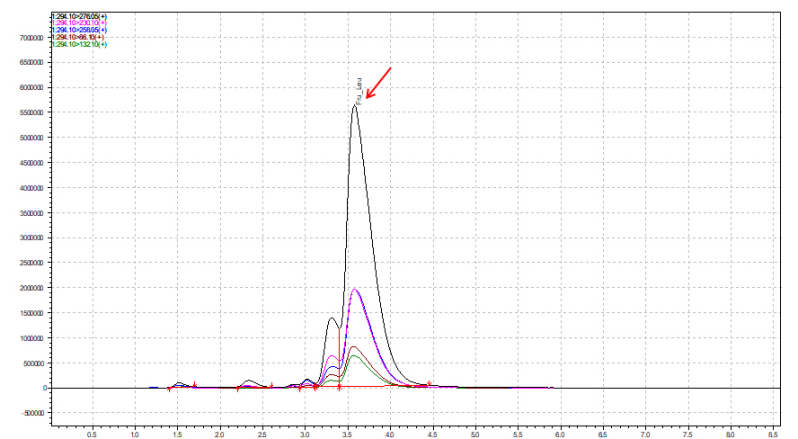


**S3 Fig. Analysis of effective indicator components (FL) of elderberry extract by** **multiple reaction monitoring mass spectrometry. FL, fructose–leucine.**
