# Supplementary material for: Ameliorative effects of elderberry (Sambucus nigra L.) extract and extract-derived monosaccharide-amino acid on H2O2-induced decrease in testosterone-deficiency syndrome in a TM3 Leydig cell
Source: PLoS One. 2024 Apr 25;19(4):e0302403. doi: 10.1371/journal.pone.0302403 (PMC11045058; doi:10.1371/journal.pone.0302403)
Supplement: S1 Table — (DOCX) [file pone.0302403.s004.docx]

**S1 Table. Elderberry extraction yield.**

| **No.** | **Elderberry weight (kg)** | **Powder weight (kg)** | **Yield (%)** |
| --- | --- | --- | --- |
| 1 Lot | 70 | 21 | 30.00 |
| 2 Lot | 70 | 30 | 42.80 |
| 3 Lot | 70 | 32 | 45.70 |

All the processes were performed in a GMP production facility (Medience Co., Ltd., Gangwon, Chuncheon-si, Korea).
