# Supplementary material for: Ameliorative effects of elderberry (Sambucus nigra L.) extract and extract-derived monosaccharide-amino acid on H2O2-induced decrease in testosterone-deficiency syndrome in a TM3 Leydig cell
Source: PLoS One. 2024 Apr 25;19(4):e0302403. doi: 10.1371/journal.pone.0302403 (PMC11045058; doi:10.1371/journal.pone.0302403)
Supplement: S2 Table — (DOCX) [file pone.0302403.s005.docx]

**S2 Table. Chemical analysis of the powdered elderberry extract.**

| **Test item** | **Standard** | **Results** |
| --- | --- | --- |
| Appearance | Unique color and taste, no strange tasting or smelling. | Suitable |
| Moisture | 5.0% below | 1.7% |
| Foreign materials test | N.D | N.D |
| Coliforms | Negative | Negative |
| Total colony counts | 10,000 cfu/g below | 0 cfu/g |

This test was conducted by the korea functional food research center(Seongnam-si, Gyeonggi-do, korea). N.D: Non-detected.
