# Supplementary material for: Ameliorative effects of elderberry (Sambucus nigra L.) extract and extract-derived monosaccharide-amino acid on H2O2-induced decrease in testosterone-deficiency syndrome in a TM3 Leydig cell
Source: PLoS One. 2024 Apr 25;19(4):e0302403. doi: 10.1371/journal.pone.0302403 (PMC11045058; doi:10.1371/journal.pone.0302403)
Supplement: S3 Table — (DOCX) [file pone.0302403.s006.docx]

**S3 Table. Nutritional Analysis of the Powdered Elderberry Extract.**

| **Test item** | **Unit** | **Results** |
| --- | --- | --- |
| Calories | Kcal/100g | 358.26 |
| Carbohydrates | % | 83.33 |
| Crude protein | % | 6.10 |
| Crude fat | % | 0.06 |
| Sodium | mg/100g | 57.32 |

This test was conducted by the korea functional food research center(Seongnam-si, Gyeonggi-do, korea). N.D: Non-detected.
