# Supplementary material for: Ameliorative effects of elderberry (Sambucus nigra L.) extract and extract-derived monosaccharide-amino acid on H2O2-induced decrease in testosterone-deficiency syndrome in a TM3 Leydig cell
Source: PLoS One. 2024 Apr 25;19(4):e0302403. doi: 10.1371/journal.pone.0302403 (PMC11045058; doi:10.1371/journal.pone.0302403)
Supplement: S4 Table — (DOCX) [file pone.0302403.s007.docx]

**S4 Table. Safety analysis of elderberry extract powder.**

| **Test item** | | **1Lot** | **2Lot** | **3Lot** |
| --- | --- | --- | --- | --- |
| Micro biological test | *E. coli* | 0 CFU/g | 0 CFU/g | 0 CFU/g |
|  | Coliform | 0 CFU/g | 0 CFU/g | 0 CFU/g |
|  | Total colony counts | 0 CFU/g | 0 CFU/g | 0 CFU/g |
|  | *Staphylococcus aureus* | 0 CFU/g | 0 CFU/g | 0 CFU/g |
|  | Yest and mold | 0 CFU/g | 0 CFU/g | 0 CFU/g |
|  | *Salmolella spp.* | Negative | Negative | Negative |
| Heavy metal | Cadmium | 0.0 mg/kg | 0.0 mg/kg | 0.0 mg/kg |
|  | Lead | 0.0 mg/kg | 0.0 mg/kg | 0.0 mg/kg |
|  | Mercury | 0.0 mg/kg | 0.0 mg/kg | 0.0 mg/kg |
|  | Arsenic | N.D | N.D | N.D |
| Pesticide residues (320 Items) | | N.D | N.D | N.D |

This test was conducted by the korea functional food research center(Seongnam-si, Gyeonggi-do, korea). N.D: Non-detected. N.D: Non-detected.
