# Supplementary material for: Ameliorative effects of elderberry (Sambucus nigra L.) extract and extract-derived monosaccharide-amino acid on H2O2-induced decrease in testosterone-deficiency syndrome in a TM3 Leydig cell
Source: PLoS One. 2024 Apr 25;19(4):e0302403. doi: 10.1371/journal.pone.0302403 (PMC11045058; doi:10.1371/journal.pone.0302403)
Supplement: S5 Table — (DOCX) [file pone.0302403.s008.docx]

**S5 Table. Analysis of rutin content as an indicator of elderberry extract.**

| **No.** | **Rutin (mg/g)** |
| --- | --- |
| 1 Lot | 2.66 |
| 2 Lot | 2.92 |
| 3 Lot | 2.63 |
| Mean | 2.74 |

This test was conducted by the korea functional food research center(Seongnam-si, Gyeonggi-do, korea).
