# Supplementary material for: Ameliorative effects of elderberry (Sambucus nigra L.) extract and extract-derived monosaccharide-amino acid on H2O2-induced decrease in testosterone-deficiency syndrome in a TM3 Leydig cell
Source: PLoS One. 2024 Apr 25;19(4):e0302403. doi: 10.1371/journal.pone.0302403 (PMC11045058; doi:10.1371/journal.pone.0302403)
Supplement: S6 Table — (DOCX) [file pone.0302403.s009.docx]

**S6 Table. Analysis of the content of Fructose-leucine in the extract of elderberry.**

| **No.** | **Fructose-leucine (mg/g)** |
| --- | --- |
| 1 Lot | 4.99 |
| 2 Lot | 5.05 |
| 3 Lot | 5.23 |

This test was conducted by the korea functional food research center(Seongnam-si, Gyeonggi-do, korea).
