# Supplementary material for: Ameliorative effects of elderberry (Sambucus nigra L.) extract and extract-derived monosaccharide-amino acid on H2O2-induced decrease in testosterone-deficiency syndrome in a TM3 Leydig cell
Source: PLoS One. 2024 Apr 25;19(4):e0302403. doi: 10.1371/journal.pone.0302403 (PMC11045058; doi:10.1371/journal.pone.0302403)
Supplement: S7 Table — (DOCX) [file pone.0302403.s010.docx]

**S7 Table. Antibody information**

| Name of antibody | Host Animal | Molecular weight  (kDa) | Dilution for Western blot | Distributor, Cat. Num. |
| --- | --- | --- | --- | --- |
| Star | Mouse/Mono | 30 | 1:1000 | Santa Cruz Biotechnology,  SC-166821 |
| 3β-hsd | Mouse/Mono | 42 | 1:1000 | Santa Cruz Biotechnology,  SC-515120 |
| 17β-hsd | Mouse/Mono | 35 | 1:1000 | Santa Cruz Biotechnology,  SC-373902 |
| Cyp11a1 | Rabbit/Mono | 60 | 1:1000 | Abcam, ab272494 |
| Cyp17a1 | Rabbit/Mono | 57 | 1:1000 | Abcam, ab134910 |
| Cyp19a1 | Rabbit/Poly | 58 | 1:500 | Invitrogen, PA1-21398 |
| β-actin | Mouse/Mono | 45 | 1:2000 | Santa Cruz Biotechnology,  SC-47778 |
