# Supplementary material for: Ameliorative effects of elderberry (Sambucus nigra L.) extract and extract-derived monosaccharide-amino acid on H2O2-induced decrease in testosterone-deficiency syndrome in a TM3 Leydig cell
Source: PLoS One. 2024 Apr 25;19(4):e0302403. doi: 10.1371/journal.pone.0302403 (PMC11045058; doi:10.1371/journal.pone.0302403)
Supplement: Attachment — Submitted filename: PONE-D-23-36301_reviewed by CHJ_231227.docx [file pone.0302403.s011.docx]

PONE-D-23-36301

Article Type: Research Article Full Title: Ameliorative effects of elderberry (Sambucus nigra L.) extract and extractderived monosaccharide-amino acid on H2O2-induced decrease in testosteronedeficiency syndrome in a TM3 Leydig cell

I am happy to review this manuscript and I do recommend about some questions.

1. Could you mark for p-value not as different letters at every graph for showing the significancy about results? The significant codes had better show the EB or FL effect on Leydig cells than the different letters.

2. In cell viability result, you investigated the result from only 24hrs. Didn't you try to detect the cell viability with other hours? Why did you decide only 24hrs results in cell viability test?

3. Your schematic representation of the effects of EB or FL in Fig.7 is good. In Fig. 7, you mentioned about CYP19, didn't you check CYP19 protein level in this study. In supplementary data, you already showed the Cyp19 mRNA levels.
