# Supplementary material for: Ameliorative effects of elderberry (Sambucus nigra L.) extract and extract-derived monosaccharide-amino acid on H2O2-induced decrease in testosterone-deficiency syndrome in a TM3 Leydig cell
Source: PLoS One. 2024 Apr 25;19(4):e0302403. doi: 10.1371/journal.pone.0302403 (PMC11045058; doi:10.1371/journal.pone.0302403)
Supplement: Attachment — Submitted filename: Response to reviewer.docx [file pone.0302403.s012.docx]

PONE-D-23-36301

답변서 “ Response to Reviewer 으로 파일명

수정된 원본 : Revised Manuscript with Track Changes'. 으로 파일명

Review #1

1. Could you mark for p-value not as different letters at every graph for showing the significancy about results? The significant codes had better show the EB or FL effect on Leydig cells than the different letters.
   Response: Thank you very much for your advice. In order to effectively show the significant difference between the groups, we used "#" or "*" to indicate the p-value in three levels according to the value of the p-value: **p<*0.05, ***p* <0.01, ****p* <0.001. The results are presented on Fig. 1, 2, 3, 4, 5 and 6. (Line 198~201)
2. In cell viability result, you investigated the result from only 24hrs. Didn't you try to detect the cell viability with other hours? Why did you decide only 24hrs results in cell viability test?

**Response:** Cell viability analysis were performed for 24 and 48 hours and the results for 48 hours cell viability were added to Figure 1. The results showed that the 24-hour and 48-hour results were similar. Therefore, subsequent experiments were analyzed using 24 hours as the baseline. (Line 214~218)

3. Your schematic representation of the effects of EB or FL in Fig.7 is good. In Fig. 7, you mentioned about CYP19, didn't you check CYP19 protein level in this study. In supplementary data, you already showed the Cyp19 mRNA levels.

**Response:** The results of the protein expression analysis of cyp19a1 are shown in Figure 6. The Western blot analysis of cyp19a1 confirmed that the expression pattern was similar to that of the *cyp19a1* mRNA. The results are presented in the Results section of this report. (Line 334~345)

Reviewer #2

This paper combines two interesting topics – male reproductive deficiency due to age related testosterone deficiency, and the advantages that come along with natural products. The study is well designed and complex. Nevertheless, there are issues that need to be addressed:

1. Since the study evaluates both the elderberry extract as well as its active component fructose-leucine, both need to be addressed in the Introduction section. Fructose-leucine is really mentioned at first in the Results. Specific information on this molecule must be provided when introducing the topic.

Response: Thank you for pointing this out. In the introduction, a description of FL has been added. (Line 87~90)

2. Overall, the Results section only serves to describe the data gathered in the experiments. All additional information, explanations, speculations should be moved either into the Introduction or the Discussion.

Response: Thank you for pointing this out. Only the analysis results are presented in the results section; other information and speculation are in the discussion section. (Line 356~362, 366~411, 435~438, 445~448, 450~460, 481~507)

3. In Material and Methods, the authors describe the preparation of the extract while at the same time informing that a readily available extract was bought. Please, clarify.

Response: The Elderberry Extract has been extracted in a GMP facility as stated, and we have removed all information related to the purchase of the extract.

4. While the supplementary files contain a description of the extract, what I am missing, is a complex chemical composition of other biologically available biomolecules present in it. HPLC has obviously been performed, however only rutin is indicated. Why was rutin selected as an indicator?

Response: We have added some experimental results to the introduction to explain why we have chosen rutin as the indicator of choice. (Line70~81)

5. What is especially important with respect to the chemical composition is that obviously, there are other molecules in the extract that may exhibit effects that are different to fructose-leucine – meaning that there are other compounds that exhibit either beneficial or adverse properties and act in synergy or antagony with fructose-leucine. This must be properly discussed in the Discussion section.

Response: Thank you for pointing this out. We've added a response to this in the discussion part. (Line 481~490)

6. Overall, the Discussion section is very short and not very well written. The authors do start with explaining the chemistry of the extract and/or fructose-leucine, however the rest of the Discussion only repeats previous studies. It should contain possible explanations to the observed results, hypotheses, speculations on the mechanisms of action supported by previous studies on the male reproductive tissues and cells. Limitations and future prospects should be addressed as well.

Response: I have expanded the discussion to include a description of what you pointed out, especially the hypotheses and speculations regarding mechanisms of action supported by previous studies on male reproductive tissues and cells. I have also added a discussion of the limitations and prospects for the future. (Line 375~384, 404~411, 450~460, 492~507)

7. The Material and Methods section is lacking. The concentrations of the extract and fructose-leucine are not specified, there is no explanation on the selected concentration range. Extracts tend not to dissolve well in culture media – were the chemicals dissolved in a solvent prior to their administration to the cells? If so, was an equal amount of the solvent added to the control group? Similarly, specify what hydrogen peroxide concentration was used and why in the material and methods section.

Response: In the Materials and Methods section, we have added the concentration ranges of the extracts, FL, and H2O2. We have also added details on the dissolution of the extracts and a section on the solvent treatment of equal amounts of the control. (Line 115~124, 128~130)

8. The cell viability and ROS assays are described very briefly – either add a relevant reference where the assays have been published or describe the assays in more detail to assure reproducibility.

Response: A detailed description of cell viability and ROS assays was provided. (Line 214~228, 262~273)

9. Please, add a supplementary table describing the antibodies in more detail: their isotype, clonality as well as concentration used and the blocking medium (milk or BSA).

Response: A descriptive supplemental table (S7 Table) for antibodies has been added. The Blocking Buffer is described in more detail. (Line 181~183)

10. On a minor note, please explain abbreviations when first used in the text.

Response: We've updated our documentation on abbreviations.

**steroidogenic acute regulatory protein (StAR)**

**cytochrome P450 11A1(CYP11A1**

**cytochrome P450 17A1(CYP17A1)**

**cytochrome P450 19A1(CYP19A1, aromatase)**

**3β-hydroxysteroid dehydrogenase (3β-HSD)**

**17β-hydroxysteroid dehydrogenase(17β-HSD)**

11. Other modification

Add content to your abstract and correct abbreviations (Line 26~29, 33~35)

Correct the notation of genes and proteins (Line 159~162, 183~192)

Adding and editing content to the conclusion (Line 520~522, 525~529)

Add references (Line 666~808)
